# Clinical Practices and Opinions toward Gastrostomy Use in Patients with Atypical Parkinsonian Syndromes: A National Survey in the UK

**DOI:** 10.1002/mdc3.14196

**Published:** 2024-08-27

**Authors:** Christopher Kobylecki, Yee Yen Goh, Rahema Mohammad, Alanna Beat, Emilia Michou, Samantha Pavey, Huw Morris, Henry Houlden, Viorica Chelban

**Affiliations:** ^1^ Division of Neuroscience, Manchester Academic Health Science Centre University of Manchester Manchester UK; ^2^ Department of Neurology, Manchester Centre for Clinical Neurosciences Northern Care Alliance NHS Foundation Trust Salford UK; ^3^ Department of Neuromuscular Diseases, Queen Square Institute of Neurology University College London London UK; ^4^ Division of Diabetes, Endocrinology and Gastroenterology University of Manchester Manchester UK; ^5^ Department of Speech and Language Therapy, School of Health Rehabilitation Sciences University of Patras Patras Greece; ^6^ Multiple System Atrophy Trust London UK; ^7^ Department of Clinical and Movement Neurosciences, Queen Square Institute of Neurology University College London London UK; ^8^ Neurobiology and Medical Genetics Laboratory “Nicolae Testemitanu” State University of Medicine and Pharmacy Chisinau Moldova

**Keywords:** gastrostomy, atypical parkinsonian syndromes, dysphagia, multiple system atrophy, progressive supranuclear palsy

## Abstract

**Background:**

Severe dysphagia poses a significant challenge for clinicians regarding feeding tube choices, practices, and timing due to a lack of evidence‐based guidance.

**Objectives:**

To assess national clinical practices and opinions on gastrostomy use in patients with atypical parkinsonian syndromes (APS) across the UK.

**Methods:**

Online survey was administered to clinicians and allied health professionals regarding availability of services, current use, perceived advantages, and problems associated with gastrostomy insertion.

**Results:**

We received responses from 47 respondents across 12 UK centers, including 44 clinicians specialized in APS. Consensus was observed regarding primary indications for gastrostomy insertion and circumstances justifying avoidance of the procedure. Limitations in recommending gastrostomy due to insufficient evidence on safety and outcomes, survival and quality of life were identified. Widespread agreement on delays in gastrostomy discussions was highlighted as a challenge in optimizing patient care, together with variability in current practices and concerns over the lack of a standardized gastrostomy pathway, emphasizing the need for further research to address existing evidence gaps.

**Conclusion:**

This multi‐center survey highlights agreement among clinicians on key aspects of indication, challenges, and limitations such as delayed decision‐making and the absence of standardized pathways regarding the timing, method, and overall approach to gastrostomy insertion in APS. This study identified next steps to facilitate a more structured approach to future research toward a consensus on best practices for gastrostomy in APS. Addressing these challenges is crucial for enhancing patient outcomes and overall care quality in APS.

Atypical parkinsonian syndromes (APS) are distinct neurodegenerative syndromes characterized by rapidly progressive parkinsonism, poor response to levodopa and other atypical clinical features such as early autonomic failure, apraxia and postural instability.^1^ APS are commonly differentiated into multiple system atrophy (MSA), progressive supranuclear palsy (PSP) and corticobasal syndrome (CBS). Although they are pathologically distinct, they share similarities in their clinical presentations.

Dysphagia in APS occurs in earlier stages of the disease compared to idiopathic Parkinson's disease (PD). The onset of dysphagia within 1 year of parkinsonism onset has very high diagnostic specificity for APS and has been associated with reduced survival.^2^ Dysphagia is a large clinical concern in APS, occurring in up to 73% of MSA patients,^3^ up to 80% of PSP patients^4^ and approximately 86% of CBS patients.^5^

Patients with severe dysphagia experience malnutrition and dehydration^6^; continued weight loss; distressing choking and coughing on attempts to swallow; prolonged and effortful mealtimes; and frequent aspiration, which increases the risk of recurrent chest infections.^7^, ^8^

In patients with pathologically confirmed APS, dysphagia was found to occur at an average of 67 months after disease onset in MSA and 42 months in PSP, compared to 130 months in idiopathic PD.^2^ Further evidence from a meta‐analysis in PSP has associated early dysphagia with reduced survival.^9^ When patients experience early symptoms of dysphagia, simple measures can be employed, such as introducing soft, moist, easy to swallow food and thickened fluids together with teaching patients safe swallowing techniques.^10^, ^11^ Usually, in severe dysphagia, enteral tube feeding becomes necessary and is frequently used as a means of safely delivering an adequate protein and calorific intake. Enteral tube feeding can be delivered either via nasogastric tube (NGT) or gastrostomy insertion. Gastrostomy is favored over NGT as problems with tube displacement or migration, nasal discomfort and poor cosmesis make NGT an inadequate long‐term solution. Gastrostomy is a medical procedure that involves cutting a small hole in the stomach wall to create an artificial opening. A flexible feeding tube is then inserted in this opening with one end lying directly in the stomach, and the other in an opening in the abdomen.^12^ There are various types of feeding tubes which may be available to patients, including percutaneous endoscopic gastrostomy (PEG), radiologically inserted gastrostomy (RIG), and peroral image‐guided gastrostomy (PIG).^13^ Gastrostomy is typically used and recommended when patients experience severe dysphagia with the goal of relieving swallowing difficulties by adding a feeding tube.^14^

The data are limited when it comes to guiding clinical practice in the management of swallowing problems in APS. Recent nutritional guidelines have recommended the discussion of gastrostomy in motor neuron disease (MND) as evidenced by the impact of this procedure on quality of life rather than survival.^15^ Currently, optimization of dopaminergic therapy and screening for nutrition and swallowing status are recommended in PD,^15^ however the rarity and lack of evidence for the safety and efficacy of gastrostomy in APS prevents an evidence‐based recommendation being made.

Gastrostomy surgery in patients with PD and APS has been associated with median survival at 400 days in PSP patients and 422 days in MSA patients with a 30‐day mortality rate of 10% in PSP.^16^ The most common adverse effect from the surgery was aspiration pneumonia. In a review study of patients with PD and related disorders, multiple sclerosis and MND, 88% experienced a complication secondary to their gastrostomy surgery.^17^ In PSP, PEG insertion has been associated with a worse disease prognosis^18^ or no evidence to suggest an improved survival in patients with PSP and CBS with advanced dementia and dysphagia.^19^ Recently, a longitudinal cohort study in APS showed that median survival post‐gastrostomy was 24 months compared with 12 months where gastrostomy was recommended but not performed (*P* = 0.008), but this was not significant when correcting for age and duration of symptoms at the time of procedure or recommendation.^20^ However, none of the studies on gastrostomy insertion on APS has assessed the impact of the timing of the procedure and the impact of the procedure on quality of life in these patients.

Recommendations for dysphagia management in APS suggest taking a speech therapy approach and considering PEG when there is an increased risk of aspiration.^21^ Further recommendations include addressing appropriate dietary consistencies and making behavioral adjustments at mealtimes. Again, PEG is recommended for proactive consideration early in the disease progression instead of after an incident of choking or aspiration. This would allow time for adequate consideration and informed decisions on the procedure in advance of necessitating intervention. Consensus for managing dysphagia in MSA, recommend dietary modifications or therapeutic swallowing measures to reduce aspiration, as well as open discussions on PEG.^8^, ^22^, ^23^ Similarly, consensus on managing dysphagia in PSP suggests early swallow therapy and key diet modifications including soft blending foods, reducing bite sizes and avoiding mixed consistencies.^24^ Further, gastrostomy should be discussed when eating becomes unsafe or when nutritional guidelines are not being satisfied.^25^ In CBS, there is similar consensus in recommending therapy and dietary changes, specifically with regards to food consistency, as well as gastrostomy discussions from early on.^26^, ^27^

Given the prevalence of dysphagia in patients with APS, gastrostomy is often discussed in clinic or considered as an option. However, the evidence for this intervention is limited. More specifically, there is no evidence on the benefits of gastrostomy in APS, and the limited available data on insertion of gastrostomy in late disease stages suggests a high mortality associated with the procedure, presenting clinicians with a serious challenge regarding feeding tube choices, practices, and timing and requiring them to draw on incomplete and sometimes conflicting evidence, personal experience, and compassion to deliver supportive patient‐centered care. However, obtaining a consensus and gathering evidence on gastrostomy insertion in APS may help with advanced care planning, which is a key component in the management of these conditions.^28^ Such work has also been important to establish pathways for gastrostomy insertion in conditions such as MND.^13^

## Methods

A survey was developed using the literature review, discussions between the research team, speech and language therapists and MSA specialist nurses. The survey was designed to assess the current clinical practice for gastrostomy insertion in patients with atypical parkinsonism, specifically MSA, PSP, and CBS. Content validity was established after review by independent movement disorder specialists and research team and the feedback was incorporated into the final version.

The online survey designed using Qualtrics had single, multiple choice‐based questions and options for free‐text comments, and a response was mandatory for all items to avoid missing data. The survey was piloted with dietitians, physicians, and clinical nurse specialists with minor changes made before distribution. Recruitment and data collection occurred between January and April 2023.

Movement disorder specialists (consultant, neurologist clinical fellows) as well as general neurologists from general district hospitals who are managing patients with APS, were invited to complete an online questionnaire (Table 1).

**TABLE 1:** Key questions on the current clinical practice of gastrostomy in atypical parkinsonian syndromes (APS)^a^

Area	Key question	Response options
Clinician background	Q1. In which region are you based?	London; Southeast England; South West England; West Midlands; East England; East Midlands; North West England; Yorkshire; North East England; Wales; Scotland; Northern Ireland
	Q2. What is your role?	Consultant; Specialist Trainee; AHP; Specialist Nurse; Other (specify)
	Q3. What is your area of expertise?	Movement disorders; General Neurology; Other (specify)
APS conditions	Q4. Do you see patients with the following conditions in your service?^a^	Multiple System Atrophy; Progressive Supranuclear Palsy; Corticobasal Syndrome
Gastrostomy procedures	Q5. In your area, where are gastrostomy procedures for patients with atypical parkinsonism performed?	Regional neurology center; District general hospitals; Both; Other (specify)
	Q6. Which methods of gastrostomy insertion are in use in your service for atypical parkinsonism?^a^	PEG; RIG; PIG; Other (specify)
	Q7. Does your unit have a pathway for gastrostomy insertion in atypical parkinsonism?	Yes; No; Don't know
Timing of initiating gastrostomy	Q8. In your opinion, in patients with MSA, gastrostomy should be offered?^a^	See Table 2
	Q9. In your opinion, in patients with PSP/CBS gastrostomy should be offered?^a^	See Table 2
Limitations in recommending gastrostomy	Q10. Which of the following factors limit your ability to recommend gastrostomy insertion in atypical parkinsonian syndromes (APS)?^a^	See Table 3

^a^ Questions allowing for multiple responses.

The speech and language therapists and MSA specialist nurses and physicians answered questions regarding current availability of services for gastrostomy assessment and interventions. Questions regarding current use, perceived advantages, and problems of gastrostomies were answered by physicians.

Quantitative data consisted entirely of categorical variables expressed as frequencies and percentages. Comparisons between clinician's responses were analyzed using Fisher's exact test due to low expected cell counts.

## Results

### Respondent Demographics

Forty‐seven clinicians managing patients with atypical parkinsonian syndromes completed the survey (see Table 1). Clinicians were recruited from 11 regions in the UK including London, South‐East England, South‐West England, Midlands, North‐West England, North‐East England, Scotland, Wales, and Northern Ireland. Among the respondents, 70% were consultants, 17% were MSA specialist nurses, 9% were neurologist specialist trainees, and 4% were allied health professionals (AHPs). Most respondents were working in movement disorders specialist clinics (81%), 15% in general neurology clinics involved in APS patient care and 4% in other areas.

Within atypical parkinsonism, 94% of clinicians reported managing patients with PSP, 89% managing MSA patients and 85% managing patients with CBS.

### Availability of Gastrostomy Procedures and Clinical Pathways for these Interventions in MSA, PSP, and CBS

The survey identified that all methods for gastrostomy insertion (PEG, RIG, and PIG) were available for patients with APS. Data on the availability of these methods was completed by 44 respondents. PEG is the most commonly available method of gastrostomy insertion for APS patients, being offered in all 11 regions assessed in this survey and is the only available option in two of the regions. PEG and RIG are available methods in six of the 11 regions surveyed. Interestingly, only three regions offer all three (PEG, RIG and PIG) methods of gastrostomy insertion for APS patients.

Gastrostomy procedures for patients with APS were reported to be performed in both regional neurology centers and district general hospitals. Although at least one or more options for gastrostomy procedures are available at every site involved in the survey, 70% of clinicians reported that their unit does not have a pathway for gastrostomy insertion in APS and only 20% of clinicians stated their unit does have a pathway while 10% were unsure whether a pathway existed or not.

### Choice of Timing for Gastrostomy Insertion in MSA, PSP, and CBS

The remainder of the survey was directly addressed to clinicians who had the responsibility to take the decision on whether a gastrostomy should be recommended or not (41 clinician respondents).

Firstly, they were asked what criteria influence the choice of timing for offering gastrostomy to patients with MSA (Table 2). Very few clinicians reported that gastrostomy should be offered at the time of or shortly after MSA diagnosis (5%, 2 out of 41). Most clinicians reported that gastrostomy should be offered when there have been recurrent instances of chest infections (76%, 31 out of 41); when patients present with symptoms of persistent dysphagia (63%, 26 out of 41); and when there has been more than 10% weight loss from baseline over 6 months (63%, 26 out of 41). Other cited circumstances for gastrostomy recommendation included: at the time of onset of aspiration pneumonias; when BMI drops to less than 18.5 kg/m^2^; and when patients begin experiencing more difficulty with meals, particularly if patients are taking an extended period to eat. The criteria that influence the choice of timing for offering gastrostomy to patients with PSP or CBS are also shown in Table 2. A few clinicians reported that gastrostomy should be a discussion early on, ideally shortly after diagnosis (8%, 3 out of 38), but emphasizing that it need not necessarily be offered then. Similarly, to patients with MSA, the majority of clinicians reported that gastrostomy should be offered when recurrent chest infections have occurred (74%, 28 out of 38); when weight loss is more than 10% from baseline over 6 months (68%, 26 out of 38); when there are symptoms of persistent dysphagia (66%, 25 out of 38); and when aspiration is identified (63%, 24 out of 38). Other criteria were that it should be offered when there are intermittent symptoms of dysphagia; and when there is increased difficulty with meals, particularly if patients are taking an extended period to eat.

**TABLE 2:** Main criteria influencing the timing of gastrostomy recommendation from the perspectives of clinicians working with patients with MSA and PSP/CBS

Gastrostomy should be offered to patients:	In MSA. Frequency from 41 respondents (%)	In PSP or CBS. Frequency from 38 respondents (%)
When recurrent chest infections have occurred	31 (76%)	28 (74%)
Weight loss of >10% from baseline over 6 months	26 (63%)	26 (68%)
When presenting with symptoms of persistent dysphagia	26 (63%)	25 (66%)
When aspiration identified eg, at video fluoroscopy	24 (59%)	24 (63%)
BMI <18.5 kg/m^2^	20 (49%)	20 (53%)
When experiencing more difficulty with meals	16 (39%)	15 (39%)
When presenting with symptoms of intermittent dysphagia	14 (34%)	11 (29%)
Clinical or biochemical evidence of dehydration	11 (27%)	8 (21%)
At or shortly after diagnosis	2 (5%)	3 (8%)
Any other criteria for offering gastrostomy?	1 (2%) Patient request	4 (11%) Patient request Taking too long to eat meals Distress from eating
Any situations where gastrostomy should be avoided?	8 (20%) Patient declined the procedure. Unlikely to improve quality of life. Disease is too severe. Major prior abdominal surgery.	13 (34%) Advanced cognitive decline or dementia. Disease is too severe. Patient declined the procedure. Unlikely to improve quality of life. Major prior abdominal surgery.

Abbreviations: CBS, corticobasal syndrome; MSA, multiple system atrophy; PSP, progressive supranuclear palsy.

#### Decision‐Making Criteria for Avoiding Gastrostomy Insertion in MSA, PSP, and CBS

Then clinicians identified instances where, in their expertise, gastrostomy insertion in MSA, PSP and CBS should be avoided. In MSA, the most frequent contraindications to recommending a gastrostomy were: patients expressed that they don't want a gastrostomy (5%, 2 out of 41); when it was assumed that its insertion would be unlikely to improve overall health and quality of life (5%, 2 out of 41). Other reasons included: the disease is too advanced (2%, 1 out of 41); patients have poor pre‐morbid status (2%, 1 out of 41) and if the patient has had previous major abdominal surgery (2%, 1 out of 41). Clinicians also identified instances where, in their expertise, gastrostomy insertion in PSP and CBS should be avoided. Reasons included when dementia is present (5%, 2 out of 38); where there is early advanced and significant cognitive impairment (5%, 2 out of 38); when patients have requested not to have the surgery (3%, 1 out of 38); and when the surgery has little chance of improving quality of life (5%, 2 out of 38).

### Limitations in Recommending Gastrostomy Insertion in MSA, PSP, and CBS

The survey asked clinicians to report on what factors limit their ability to recommend gastrostomy insertion in atypical parkinsonian syndromes. The majority (68%, 26 out of 38) reported the lack of supporting evidence on the safety, survival and/or quality of life outcomes after gastrostomy insertion (Table 3). Many (61%, 23 out of 38) reported that the decisions and discussions on gastrostomy insertion are delayed until the advanced stage of disease precluding the intervention together with a lack of local or national care pathways for gastrostomy insertion in APS (55%, 21 out of 38). Finally, a small number of clinicians (13%, 5 out of 38) reported that there is a lack of local services offering gastrostomy insertion to patients. In free‐text responses, additional barriers in the ability to recommend gastrostomy included that there should be an established multi‐disciplinary team for handling gastrostomy intervention, consisting of a gastroenterologist, dietician, SALT and neurologist.

**TABLE 3:** Main limitations reported by clinicians when recommending gastrostomy for patients with atypical parkinsonian syndromes (APS)

Limitations in recommending gastrostomy in APS	Frequency from 38 respondents (%)
Lack of local services offering gastrostomy insertion.	5 (13%)
Lack of local or national care pathways for gastrostomy insertion in APS.	21 (55%)
Decisions/discussion on gastrostomy insertion delayed until advanced stage precluding intervention.	23 (61%)
Lack of supporting evidence on safety, survival and/or quality of life outcomes after gastrostomy insertion.	26 (68%)

## Discussion

Despite the high prevalence and severity of dysphagia in APS, understanding of gastrostomy use in this context remains limited, with little evidence available to guide optimal timing or preferred insertion methods. This study reflects the clinician perspectives on gastrostomy procedures and their management in current UK clinical practice. Our findings revealed a lack of clear pathways for gastrostomy insertion across most sites, despite the availability of various insertion methods (PEG, PIG, and RIG).

However, consensus emerged among clinicians regarding criteria warranting gastrostomy, primarily driven by recurrent chest infections, persistent dysphagia, and aspiration risk. Conversely, reasons to avoid gastrostomy, including patient refusal and perceived limited enhancement of quality of life, were acknowledged. Notably, concerns persist regarding the procedure's safety and efficacy due to insufficient evidence, with delayed timing of procedural discussions identified as a significant challenge in optimizing patient care. Data from other similarly rapidly progressing neurodegenerative conditions such as MND show that offering gastrostomy to MND patients as close to the first appearance of their bulbar symptoms has been found to have potential advantages for preventing dehydration and malnutrition, as well as reducing the risks of aspiration.^13^, ^29^ Furthermore, clinical guidance for MND has recommended discussing gastrostomy early in the disease course, combined with a timely initiation of the intervention. This guidance suggests early interventions have the potential to reduce complications in surgery, promote better outcomes, extend survival, and address the negative effects of functional losses on quality of life.^30^ Interestingly, despite the broad consensus on indications for consideration of gastrostomy, our recent research in the PROSPECT‐M‐UK dataset suggested that gastrostomy was offered in only around 13% of the total APS population and performed in 6%.^20^ The lack of pathways for gastrostomy insertion and other barriers to implementation identified in this survey may explain this apparent discrepancy. However, unlike in MND, in APS conditions the risk of aspiration from saliva or regurgitated feed is maintained even after gastrostomy insertion, therefore this aspect should be considered when gastrostomy is being discussed.

While evidence on gastrostomy's impact on quality of life in APS remains scarce, insights from other conditions like MND suggest potential benefits. Studies have indicated improved daily living and nutritional status post‐gastrostomy, despite limited effects on survival rates. Patients have expressed a relief of anxiety following their PEG surgery as they no longer worry about effortful mealtimes, dehydration, malnutrition, and aspiration risks.^31^, ^32^ Similarly, in MND, gastrostomy discussions for a large proportion of patients happen early after diagnosis before there is an urgent need to decide. For others, gastrostomy is mainly discussed when there is a clear indication for it, ie, when there is severe dysphagia, weight loss and prolonged mealtimes.^33^ Evidence based recommendations for ALS further suggest the need for early gastrostomy soon after the onset of dysphagia to have the greatest benefit to quality of life.^32^ In the APS conditions, additional considerations need to be taken into account when deciding on the timing for this conversations. Communication difficulties for MSA patients and cognitive impairment in PSP and CBS may impact patients’ ability to fully describe their wishes in later stages of the conditions therefore more detailed studies are needed in the future to address these issues.

Although this study succeeds in gathering information on gastrostomy use in APS, it is not without limitation. Firstly, this is a small sample size of only one representative per center. This may pose an issue in terms of representing individual views rather than representing entire center views. However, attempts were made to reduce individual bias as participants were requested to reflect on shared perspectives rather than just their own. Furthermore, this study was based on a survey and not on clinical observation. There may be differences in actual clinical practice and what is reported via survey. As a first step, this survey involved only neurologists, however further engagement with a broader group of specialists including but not limited to palliative care, geriatricians, gastroenterologists and neuro‐radiologists is needed to determine the wider clinical practice, perceived limitations and identify the needs to appropriate delivery of gastrostomy insertion in these patients’ groups. Finally, this survey was bespoke, not validated for use and does not encompass the full scope of nutritional practices in relation to APS.

APS care in the UK is largely delivered by a network of multidisciplinary specialist clinics supported by specialist nurses, which provides an excellent opportunity to prospectively evaluate gastrostomy practice and develop evidence‐based guidelines for gastrostomy placement in these conditions. This is in line with the International Consensus Conference call for more research on indication, optimal timing, and method of gastrostomy in patients with MSA.^8^

In conclusion, this study is the first step to address the knowledge gap surrounding feeding tube choices by ascertaining the current standard of practice and provides valuable insights into clinician perspectives on gastrostomy use in APS in the UK. It highlights a consensus on views regarding indications for and concerns related to the intervention, but most importantly, it shows significant gaps in understanding and the lack of consensus regarding the timing, method, and overall approach to gastrostomy in APS patients despite the acknowledged high prevalence of dysphagia and related complications. While clinicians generally agree on the criteria warranting gastrostomy—such as recurrent chest infections, persistent dysphagia, and aspiration risk—there is no clear, standardized pathway for its implementation across UK clinical practices. This study identified several next steps that are necessary to address these gaps and to establish a consensus on best practices for gastrostomy in APS. Firstly, engaging a broader group of specialists, including palliative care, geriatricians, gastroenterologists, and neuro‐radiologists, to collaborate with neurologists in developing comprehensive, evidence‐based guidelines for gastrostomy in APS. This should include clear criteria for timing, method selection (PEG, PIG, and RIG), and patient management post‐insertion. Then, conduct prospective studies within the UK's multidisciplinary specialist clinics network to gather robust data on gastrostomy outcomes in APS. This data should inform the creation of standardized care pathways and improve decision‐making processes, establish protocols for early and regular discussions about gastrostomy with APS patients, considering communication difficulties in MSA and cognitive impairments in PSP and CBS in later stages of disease. This approach, like the one recommended for MND patients, should aim to address patient concerns early and reduce the risks associated with delayed decision‐making. Lastly, we incurrage to implement approaches that prioritize research that specifically investigates the safety and efficacy of gastrostomy in APS, considering the unique challenges with these conditions.

## Author Roles

(1) Research project: A. Conception, B. Organization, C. Execution; (2) Analysis and interpretation of data: A. Design, B. Execution, C. Review and Critique; (3) Manuscript Preparation: A. Writing of the first draft, B. Review and Critique.

C.K.: 1A, 1B, 1C

Y.Y.G.: 1A, 2A, 2B, 2C, 3B

R.M.: 2A, 2B, 2C, 3B

A.B.: 2B, 2C, 3B

E.M.: 2A, 2B, 2C, 3B

S.P.: 2A, 2B, 2C, 3B

H.M.: 2C, 3B

H.H.: 2C, 3B

V.C.: 1A, 1B, 1C, 2A, 2B, 2C, 3A, 3B

## Disclosures

**Ethical Compliance Statement:** This study was approved by the London – Queen Square Research Ethics Committee (14/LO/1575). Informed patient consent was not necessary for this work. We confirm that we have read the Journal's position on issues involved in ethical publication and affirm that this work is consistent with those guidelines.

**Funding Sources and Conflicts of Interest:** This study was supported by the MSA Trust (Grant 576402). We are grateful to the MSA Coalition, Medical Research Council (MRC UK MR/J004758/1, G0802760, G1001253), The Wellcome Trust equipment and strategic awards (Synaptopathies) (WT093205MA and WT104033/Z/14/Z), and the PSP Association for their support. VC received grants from the Multiple System Atrophy Trust/ABN Clinical Research Training fellowship Grant F84 ABN 540868, Multiple System Atrophy Trust (PROSPECT‐M‐UK Project), Multiple System Atrophy Coalition Grant 567,540, The Guarantors of Brain Grant 565,908 and King Baudouin Foundation/ Sophia Fund. The authors report no conflicts of interest related to this work. The authors alone are responsible for the content and writing of the paper.

**Financial Disclosures for the Previous 12 Months:** The authors declare that there are no additional disclosures to report.

## Data Availability

The data that support the findings of this study are available from the corresponding author upon reasonable request.
